# Fnr and ArcA Regulate Lipid A Hydroxylation in *Salmonella* Enteritidis by Controlling *lpxO* Expression in Response to Oxygen Availability

**DOI:** 10.3389/fmicb.2018.01220

**Published:** 2018-06-08

**Authors:** Paulina A. Fernández, Felipe Velásquez, Héctor Garcias-Papayani, Fernando A. Amaya, Jaime Ortega, Sebastián Gómez, Carlos A. Santiviago, Sergio A. Álvarez

**Affiliations:** Laboratorio de Microbiología, Departamento de Bioquímica y Biología Molecular, Facultad de Ciencias Químicas y Farmacéuticas, Universidad de Chile, Santiago, Chile

**Keywords:** *Salmonella*, LPS, lipid A hydroxylation, anaerobiosis, LpxO, Fnr, ArcA

## Abstract

Lipid A is the bioactive component of lipopolysaccharide, and presents a dynamic structure that undergoes modifications in response to environmental signals. Many of these structural modifications influence *Salmonella* virulence. This is the case of lipid A hydroxylation, a modification catalyzed by the dioxygenase LpxO. Although it has been established that oxygen is required for lipid A hydroxylation acting as substrate of LpxO in *Salmonella*, an additional regulatory role for oxygen in *lpxO* expression has not been described. The existence of this regulation could be relevant considering that *Salmonella* faces low oxygen tension during infection. This condition leads to an adaptive response by changing the expression of numerous genes, and transcription factors Fnr and ArcA are major regulators of this process. In this work, we describe for the first time that lipid A hydroxylation and *lpxO* expression are modulated by oxygen availability in *Salmonella enterica* serovar Enteritidis (*S*. Enteritidis). Biochemical and genetic analyses indicate that this process is regulated by Fnr and ArcA controlling the expression of *lpxO*. In addition, according to our results, this regulation occurs by direct binding of both transcription factors to specific elements present in the *lpxO* promoter region. Altogether, our observations revealed a novel role for oxygen acting as an environment signal controlling lipid A hydroxylation in *S*. Enteritidis.

## Introduction

*Salmonella enterica* serovar Enteritidis (*S*. Enteritidis) is considered the leading cause of food-borne salmonellosis worldwide (Roberts and Sockett, 1994; Guard-Petter, 2001). In humans, this pathogen causes a self-limited gastroenteritis characterized by diarrhea, fever, and abdominal pain (Guard-Petter, 2001). Virulence mechanisms used by *S*. Enteritidis are poorly understood, and most knowledge on the subject is based on studies carried out in *S*. Typhimurium, which also causes gastroenteritis in humans. In spite of this, it is known that *S*. Enteritidis lipopolysaccharide (LPS) plays a major role in pathogenicity, as genes involved in the synthesis of this macromolecule are required for systemic colonization (Silva et al., 2012; Coward et al., 2013), resistance to serum (Bravo et al., 2008), and survival in egg albumen (Coward et al., 2013).

Lipid A is the bioactive component of LPS and constitutes the anchor portion of this molecule to the outer membrane (Raetz and Whitfield, 2002). When particular structures of lipid A are recognized by the TLR-4/MD2 complex, an inflammatory response is triggered in macrophages (Park et al., 2009). In response to environmental cues, lipid A can be covalently modified by different enzymes in a process that is essential for bacterial adaptation to its host (Raetz et al., 2007). These modifications are implicated in cationic antimicrobial peptides (CAMPs) resistance and in avoiding its recognition by TLR-4.

LpxO is a Fe^2+^/α-ketoglutarate-dependent dioxygenase that catalyzes the hydroxylation of lipid A by modifying the 3’-secondary myristoyl chain to generate *S*-2-hydroxymiristate in *S*. Typhimurium (Gibbons et al., 2000, 2008). Studies addressing the role played by this modification in *Salmonella* virulence are scarce. Despite this, it has been reported that an appropriate lipid A hydroxylation level is crucial for *S*. Typhimurium replication in macrophages and for systemic colonization in mice (Moreira et al., 2013). Also, a role for lipid A hydroxylation on reduction of inflammatory response and CAMP resistance has been described in *Klebsiella pneumoniae* (Llobet et al., 2015; Mills et al., 2017).

The expression of most genes encoding lipid A-modifying enzymes is regulated by two-component systems PhoP/PhoQ and PmrA/PmrB in *S*. Typhimurium (Raetz, 2001). PhoP/PhoQ responds to environmental signals encountered by *S*. Typhimurium and *S*. Enteritidis during infection and is essential for virulence of these bacteria in mice (Dalebroux and Miller, 2014). In addition, PhoP/PhoQ and PmrA/PmrB systems are functionally linked (Gunn et al., 2000). Although little is known on the mechanisms that regulate *lpxO* expression in *Salmonella*, a study confirmed that this gene is not regulated by PhoP/PhoQ (Gibbons et al., 2005).

Even though it has been established that oxygen is required for lipid A hydroxylation acting as a substrate of LpxO (Gibbons et al., 2008), a regulatory role for oxygen on *lpxO* expression has not been described. The existence of such regulatory role could be relevant as low oxygen tension is one of the conditions that *Salmonella* encounters in the distal ileum and within host cells (Altier, 2005; Rhen and Dorman, 2005). Transcription factors Fnr and ArcA are the major regulators involved in adaptation to oxygen availability (Compan and Touati, 1994; Kiley and Beinert, 1998; Sawers, 1999; Fink et al., 2007; Ravcheev et al., 2007). In addition to regulating the expression of several genes involved in energy metabolism, Fnr also regulates the expression of virulence genes in *S*. Typhimurium (Fink et al., 2007). On the other hand, it has also been reported that ArcA contributes to *S*. Enteritidis virulence in the murine model (Silva et al., 2012). Previously, we have shown that Fnr and ArcA regulate O-antigen (OAg) chain-length distribution in an oxygen-dependent fashion in *S*. Enteritidis (Silva-Valenzuela et al., 2016). In the present study, we describe for the first time that lipid A hydroxylation and *lpxO* expression are regulated by oxygen availability in *S*. Enteritidis, and that Fnr and ArcA participate in this process by direct interaction with elements present in the *lpxO* promoter region.

## Materials and Methods

### Bacterial Strains, Plasmids, Media, and Growth Conditions

All bacterial strains and plasmids used in this study are listed in **Table 1**. Bacteria were routinely grown in Luria-Bertani (LB) medium (10 g/L tryptone, 5 g/L yeast extract, 5 g/L NaCl) at 37°C with agitation (180 rpm). For assays under aerobic and anaerobic conditions, overnight cultures in LB were diluted 1:100 in minimal E medium (0.2 g/L MgSO_4_ × 7H_2_O, 2 g/L citric acid monohydrate, 13.1 g/L K_2_HPO_4_ × 3H_2_O, 3.3 g/L NaNH_4_HPO_4_ × 4H_2_O, pH 7.0), supplemented with 0.2% glucose as sole carbon source. Aerobic cultures were obtained by incubation at 37°C with agitation (180 rpm) and anaerobic cultures were obtained by incubation at 37°C in an anaerobic jar with the AnaeroGen system (Oxoid). When required, media were supplemented with ampicillin (Amp, 100 mg/L), chloramphenicol (Cam, 20 mg/L), or kanamycin (Kan, 75 mg/L). Media were solidified by the addition of agar (15 g/L).

**Table 1:** Bacterial strains and plasmids used in this study.

Strain or plasmid	Relevant properties	Source or reference
***Salmonella* Enteritidis**		
NCTC13349	Wild-type strain	Laboratory stock
Δ*fnr*	NCTC13349 Δ*fnr*::FRT	Silva-Valenzuela et al., 2016
*lpxO*–*lacZ*	NCTC13349 ϕ(*lpxO*’-*lac*^+^), Kan^R^	This study
*lpxO*–*lacZ* Δ*fnr*	NCTC13349 ϕ(*lpxO*’-*lac*^+^) Δ*fnr*::*cam*, Kan^R^ Cm^R^	This study
***Escherichia coli***		
TOP10	F^-^ *mcr*A Δ(*mrr*-*hsdRMS*-*mcrBC*) ϕ80*lac*ZΔM15 Δ*lacX74 recA1 ara*Δ139 Δ(*ara*-*leu*)7697 *galU galK rpsL* (Str^R^) *endA1 nupG*	Life Technologies
**Plasmids**		
pBAD-TOPO	Cloning vector, Amp^R^	Life Technologies
parcA	*arcA* (promoter included) from *S*. Enteritidis NCTC13349 cloned in pBAD-TOPO	This study
pBAD-fnrD154A	*fnrD154A* ORF from *E. coli* K12 cloned in pBAD-TOPO	This study
pBAD-arcA	*arcA* ORF from *S*. Enteritidis NCTC13349 cloned in pBAD-TOPO	This study
pKD46	*bla* P_BAD_ *gam bet exo* pSC101 oriTS, Amp^R^	Datsenko and Wanner, 2000
pCLF4	*bla* PS1 FRT *aph* FRT P_T7_ PS2 oriR6K, Amp^R^, Kan^R^	Santiviago et al., 2009
pCLF3	*bla* PS1 FRT *cat* FRT P_T7_ PS2 oriR6K, Amp^R^, Cam^R^	Santiviago et al., 2009
pCP20	*bla cat cI*857 λP_R_ *flp* pSC101 oriTS, Cam^R^, Amp^R^	Cherepanov and Wackernagel, 1995
pKG136	*aph* FRT *lacZY*^+^ t*_his_* oriR6K, Kan^R^	Ellermeier et al., 2002
pPK822	*fnrD154A* ORF from *E. coli* K12 cloned in pUC188, Amp^R^	Lazazzera et al., 1993

### Construction of Mutant Strains

*Salmonella* Enteritidis mutant strains Δ*lpxO*::*kan* and Δ*fnr*::*cam* were constructed by the Red-swap method (Datsenko and Wanner, 2000) with modifications (Santiviago et al., 2009), using plasmid pCLF4 (Kan^R^, GenBank accession number EU629214) or pCLF3 (Cam^R^, GenBank accession number EU629213) as template. Correct allelic replacement in these mutants was confirmed by PCR amplification using primers flanking the substitution site. All primers for PCR amplifications are listed in Supplementary Table S1. Next, the mutations were transduced into the wild-type background using P22 HT105/1 *int*-201 (Schmieger, 1972) as described (Maloy, 1990). To obtain non-polar deletion mutants (i.e., Δ*lpxO*::FRT and Δ*fnr*::FRT), the antibiotic-resistance genes were removed by transforming the insertion mutants with pCP20 (Cherepanov and Wackernagel, 1995; Datsenko and Wanner, 2000).

An *lpxO*–*lacZ* transcriptional fusion was constructed using the Flp-mediated site-specific recombination method (Ellermeier et al., 2002). To do this, the Δ*lpxO*::FRT mutant harboring pCP20 was transformed with pKG136, a derivative of the *lacZ* transcriptional fusion plasmid pCE36. This procedure resulted in the stable integration of pKG136 in the chromosome and the loss of pCP20. The single-copy integration of pKG136 in the Δ*lpxO*::FRT allele was confirmed by PCR amplification as described (Ellermeier et al., 2002). Finally, the Δ*fnr*::*cam* allele was transduced into the mutant harboring the *lpxO*–*lacZ* fusion using P22 HT105/1 *int*-201.

### Lipid A Purification

Lipopolysaccharide was extracted by the TRIzol method as described (Yi and Hackett, 2000), with modifications. Bacteria from cultures grown to an OD_600_ 0.3 under aerobic or anaerobic conditions in 50 mL of minimal E medium were harvested by centrifugation. Each pellet was suspended in 2 mL of TRIzol (Life Technologies) and 400 μL of chloroform were added to the suspension. The mixture was vigorously vortexed until complete homogenization and then incubated during 30 min at room temperature. The resulting mixture was centrifuged at 3500 × *g* during 10 min at room temperature, and the aqueous phase was recovered in a clean centrifuge tube. Next, 1 mL of distilled water was added to the organic phase, the extraction procedure was repeated, and the new aqueous phase was recovered in a clean centrifuge tube. Both recovered aqueous phases were combined and lyophilized. Further LPS purification was achieved by the cold ethanol-magnesium precipitation procedure (Darveau and Hancock, 1983) followed by two rounds of Folch extraction (Folch et al., 1957). LPS integrity in our preparations was evaluated by Tricine-SDS-PAGE in 12% polyacrylamide gels (Marolda et al., 2006) and silver staining (Tsai and Frasch, 1982).

Lipid A was isolated from LPS preparations by mild acid hydrolysis as described (Caroff and Novikov, 2011), with modifications. After addition of hydrolysis solution (1% SDS in 10 mM sodium acetate pH 4.5), LPS samples were allowed to dissolve during 30 min at room temperature. Then, samples were incubated during 2 h at 100°C and the hydrolysis products were lyophilized. The sediment was suspended in 800 μL of chloroform/water/methanol (10:9:1 v/v/v) and centrifuged during 5 min at 2100 × *g*. Finally, the organic phase containing purified lipid A was recovered and the organic solvents were evaporated at room temperature. The absence of non-hydrolyzed LPS in our lipid A preparations was evaluated by Tricine-SDS-PAGE in 12% polyacrylamide gels (Marolda et al., 2006) and silver staining (Tsai and Frasch, 1982).

### MALDI-TOF Mass Spectrometry

Lipid A samples were dissolved in 90 μL of chloroform/methanol (1:1 v/v) and this solution was mixed with one volume of matrix (10 mg/mL 2.5-dihydroxybenzoic acid in 100 mM citric acid). Spectra were obtained in the negative reflection mode using a Microflex LRF mass spectrometer (Bruker Daltonics Inc., MA, United States). flexControl 3.0 (Bruker Daltonik GmbH, Germany) and mMass version 5.5.0 ^1^ software packages were used for spectrometer control and spectra analysis, respectively.

### RNA Isolation and q-RT-PCR Assays

RNA isolation was performed as described (Kroger et al., 2012) from at least three independent cultures grown in minimal E medium. Briefly, aerobic or anaerobic cultures were grown to an OD_600_ of 0.3 and total RNA was extracted using the “SV Total RNA Isolation System” (Promega). All samples were treated with RNase-free DNaseI (Qiagen) at 25°C for 45 min and kept at -20°C until further use.

For qRT-PCR assays, 1 μg of total RNA was reverse transcribed using random hexamers and SuperScript II (Life Technologies) following manufacturer’s instructions. For each gene evaluated, a specific DNA fragment (∼300 bp) was amplified using the “KAPA SYBR FAST qPCR kit Master Mix (2x) Universal” (Kapa Biosystems) as recommended by the manufacturer in an Mx3000P qPCR System with MxPro software (Agilent Technologies, Inc.). Ct values were normalized using *rpoD* as housekeeping gene.

### β-Galactosidase Assays

*Salmonella* Enteritidis strains carrying the *lpxO*–*lacZ* fusion were grown without antibiotics under aerobic or anaerobic conditions at 37°C in minimal E medium to an OD_600_ 0.3–0.5. β-galactosidase activity was measured as described (Miller, 1972). Briefly, 100 μL of culture was suspended in Z buffer pH 7.0 (0.6 M Na_2_HPO_4_, 40 mM NaH_2_PO_4_, 10 mM KCl, 1 mM MgSO_4_, 50 mM β-mercaptoethanol) to a final volume of 1 mL. Bacteria were permeabilized with 10 μL of chloroform, 10 μL of 0.1% SDS, and vortexed for 20 s. After incubation at 30°C for 10 min, 200 μL of 4 mg/mL ONPG was added to the mix. Reactions were stopped by the addition of 500 μL of 1 M Na_2_CO_3_. Each assay was made in duplicate and repeated at least three times, in different days. β-galactosidase activity was expressed in Miller units.

### Cloning of *arcA* and *fnrD154A* Alleles

For overexpression of ArcA, a DNA fragment containing the *arcA* gene (including the promoter region) was amplified from the genome of *S*. Enteritidis NCTC13349 using primers listed in Supplementary Table S1. The PCR product was purified from 1% agarose gels and cloned into pBAD-TOPO using the “pBAD-TOPO TA Expression Kit” (Invitrogen). The presence and orientation of the insert in the recombinant plasmid generated (parcA) was confirmed by PCR amplification using combinations of primers listed in Supplementary Table S1. *S*. Enteritidis strains were transformed by electroporation with plasmid parcA for ArcA overexpression assays.

For expression and purification of ArcA and FnrD154A, a DNA fragment containing the open reading frame (ORF) of *arcA* or *fnrD154A* was amplified from the genome of *S*. Enteritidis NCTC13349 and plasmid pPK822 (Lazazzera et al., 1993), respectively, using primers listed in Supplementary Table S1. Each PCR product was purified from 1% agarose gels and cloned into pBAD-TOPO to generate a 6 × His-tagged protein (C-terminal) using the “pBAD-TOPO TA Expression Kit” (Invitrogen). The presence and orientation of each insert in the recombinant plasmids generated (pBAD-arcA and pBAD-fnrD154A) were confirmed by PCR amplification using combinations of primers listed in Supplementary Table S1. Finally, *Escherichia coli* TOP10 was transformed with plasmid pBAD-arcA or pBAD-fnrD154A for expression and purification of ArcA and FnrD154A.

### Expression and Purification of ArcA and FnrD154A

Expression of ArcA and FnrD154A was induced during 4 h by adding 0.02% or 0.2% L-arabinose to cultures (OD_600_ ∼0.4) of *E. coli* TOP10 harboring pBAD-arcA or pBAD-fnrD154A, respectively. Bacteria were harvested and suspended in lysis buffer (50 mM Tris-HCl, pH 7.4, 100 mM NaCl, 500 μM DTT), supplemented with “cOmplete Mini, EDTA-free” protease inhibitor cocktail (Roche) according to manufacturer’s recommendations. Next, the mixture was supplemented with lysozyme (1 mg/mL final concentration) and MgCl_2_ (1 mM final concentration). After 30 min of incubation at 37°C, the mixture was subjected to five cycles of sonication (20 s of sonication, 1 min of recess) on ice. The lysate was centrifuged at 3500 × *g* during 5 min at 4°C, and the supernatant was recovered and further centrifuged at 21,000 × *g* during 30 min at 4°C. This clarified supernatant was recovered and supplemented with imidazole (30 mM final concentration). To purify each recombinant protein, the clarified lysate was applied to a 1 mL HisTrap FF column (GE Healthcare) previously equilibrated with 12 volumes of binding buffer (50 mM Tris-HCl pH 7.4, 500 mM NaCl, 30 mM imidazole). Then, the column was washed with 20 volumes of binding buffer. The recombinant proteins were eluted with a linear imidazole gradient (30–500 mM) in binding buffer. Purity of protein samples was checked by SDS-PAGE in 12% polyacrylamide gels stained with Coomassie Brilliant Blue G-250 (Supplementary Figure S1). Fractions containing the purified proteins were dialyzed overnight at 4°C against the corresponding reaction buffer (FnrD154A: 10 mM sodium phosphate pH 7.5, 100 mM sodium glutamate, 1 mM EDTA, 50 mM DTT, 5% glycerol; ArcA: 100mM Tris-HCl pH 8.0, 10 mM MgCl_2_, 125 mM KCl, 2 mM DTT, 10% glycerol). Protein concentration in each preparation was determined by the Bradford assay using the Coomassie Protein Assay Reagent (Thermo Scientific).

### Protein–DNA Binding Assay

Electrophoretic mobility shift assays (EMSA) were performed to evaluate protein–DNA interaction. For EMSA carried out in the presence of FnrD154A, a 480 bp fragment including the complete *lpxO* promoter region was amplified from the genome of *S.* Enteritidis NCTC13349 using primers listed in Supplementary Table S1. This fragment (120 ng) was incubated with different amounts of FnrD154A for 30 min at 37°C in reaction buffer supplemented with 50 μg BSA and 120 ng of a 261 bp fragment of non-specific competitor DNA corresponding to the polylinker region of pGEM-T Easy (Promega). Heat-denatured FnrD154A was used as negative control.

For EMSA carried out in the presence of ArcA, the complete *lpxO* promoter region was used along with internal fragments of 253, 162, and 109 bp (amplified from *S*. Enteritidis genomic DNA using primers listed in Supplementary Table S1) including unique putative ArcA binding sites (ABS) predicted in the promoter. Each fragment (120 ng) was incubated with different amounts of *in vitro* phosphorylated ArcA (P-ArcA) for 30 min at 25°C in reaction buffer. P-ArcA was obtained as described (Lynch and Lin, 1996). Non-phosphorylated ArcA and heat-denatured P-ArcA were used as negative controls.

In all cases, reaction mixtures were loaded in 6% polyacrylamide gels and resolved by electrophoresis in 0.5X TBE buffer. Gels were stained with GelRed (Thermo Fisher Scientific) 3X solution. DNA and protein–DNA complexes were detected and captured using a Syngene Dyversity Imaging System (Cambridge, United Kingdom).

## Results

### Lipid A Hydroxylation Is Modulated by Oxygen Availability in *S*. Enteritidis

**FIGURE 1:**
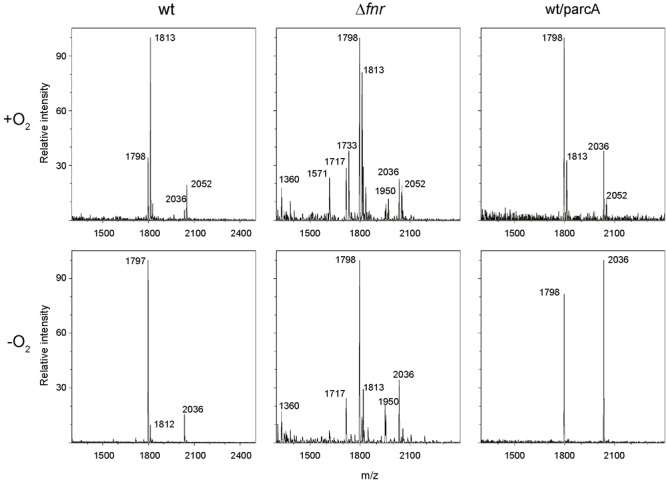
Mass spectrometry analysis of lipid A species produced by *S*. Enteritidis strains. Lipid A samples were obtained from cultures of the wild-type strain (left panels), its isogenic Δ*fnr* mutant (middle panels), and the wt/parcA strain (right panels) grown under aerobic (upper panels) or anaerobic (lower panels) conditions. MALDI-TOF mass spectrometry analysis of lipid A preparations was performed and experimental *m/z* values are indicated.

**FIGURE 2:**
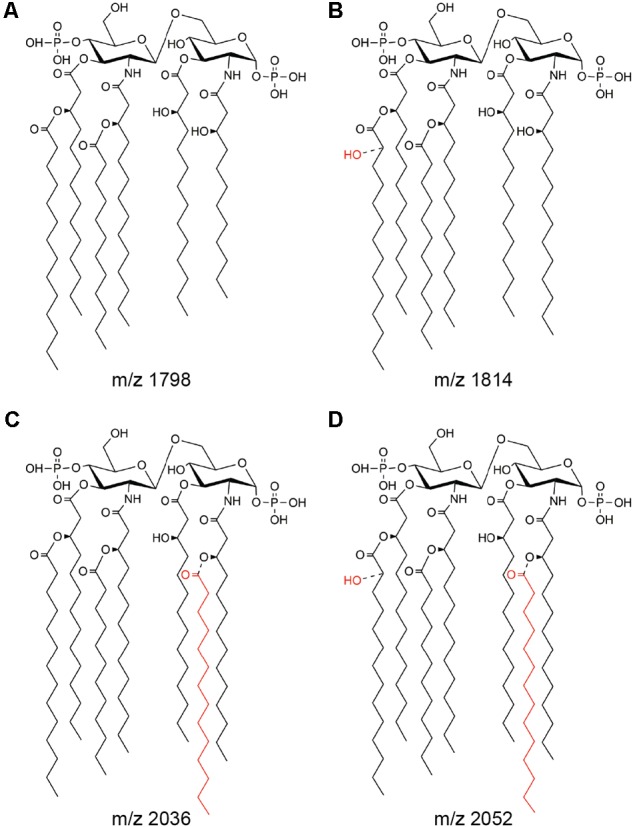
Main lipid A species produced by *S*. Enteritidis. The structures correspond to hexaacylated lipid A **(A)**, its hydroxylated counterpart **(B)**, heptaacylated lipid A **(C)**, and its hydroxylated counterpart **(D)**. *m/z* values associated to each species (Kawasaki et al., 2007) are indicated.

In order to determine the effect of oxygen availability on *S*. Enteritidis lipid A covalent modifications, the wild-type strain was grown under aerobic or anaerobic conditions and lipid A was purified and analyzed by MALDI-TOF mass spectrometry. Under aerobic conditions, the negative-ion spectrum of lipid A showed four major peaks at *m/z* 1797.5, 1813.3, 2036.1, and 2051.8 (**Figure 1**, upper left panel), which were interpreted as canonic hexaacylated lipid A, its hydroxylated counterpart, heptaacylated lipid A and its hydroxylated counterpart, respectively (**Figure 2**). Thus, hydroxylated species were more abundant than non-hydroxylated species under aerobic conditions. In contrast, non-hydroxylated species were more abundant than their hydroxylated counterparts when *S*. Enteritidis was grown under anaerobic conditions (**Figure 1**, lower left panel). Of note, during anaerobic growth, the signal corresponding to the heptaacylated hydroxylated species was undetectable. These results show that lipid A hydroxylation in *S*. Enteritidis is modulated by oxygen availability, as described in *S*. Typhimurium (Gibbons et al., 2000).

### Lipid A Hydroxylation Is Regulated by Fnr and ArcA in *S*. Enteritidis

As mentioned, oxygen is required as substrate for LpxO during lipid A hydroxylation in *S*. Typhimurium (Gibbons et al., 2000). However, a regulatory role for oxygen (acting as environmental signal) has not been described in this process. To address this possibility, we evaluated the participation of global regulators ArcA and Fnr on lipid A modification. Deletion of the *fnr* gene dramatically changed the lipid A species profile under both aerobic and anaerobic conditions, as spectra showed a higher number of species not detected in the wild-type stain (*m/z* 1360.1, 1717.4, 1733.2, and 1950.3; **Figure 1**, middle panels). These species were inferred according to published data. Thus, a peak at *m/z* 1360.1 is consistent with 3’*-O-*deacylated lipid A produced by LpxR (Reynolds et al., 2006). Peaks at *m/z* 1717.4 and 1733.2 were interpreted as lipid A dephosphorylated by UgtL (Shi et al., 2004) and its counterpart hydroxylated by LpxO (Kong et al., 2012), respectively. Although genetic evidence supports the former assignments, a peak at *m/z* 1718 (similar to 1717.4) has been interpreted as lipid A hydroxylated by LpxO, 3*-O-*deacylated by PagL, and modified with an aminoarabinose by ArnT (Guo et al., 1998). Finally, a peak at *m/z* 1950.3 is consistent with a highly modified lipid A that has been 3*-O-*deacylated by PagL, palmitoylated by PagP, hydroxylated by LpxO, and including a phosphoethanolamine attached by EptA (Kawasaki et al., 2007).

Unlike what was observed in the wild-type strain, the relative abundance of all hydroxylated lipid A species was comparable to the abundance of their non-hydroxylated counterparts when the Δ*fnr* mutant was grown under aerobic conditions (**Figure 1**, compare upper middle and upper left panels). However, under anaerobiosis, the relative abundance of hydroxylated and non-hydroxylated lipid A in the Δ*fnr* mutant was similar to that observed in the wild-type strain (**Figure 1**, compare lower middle and lower left panels). These results revealed for the first time that Fnr plays a role in the regulation of lipid A modification in *S*. Enteritidis.

As previously described, the *S*. Enteritidis Δ*arcA* mutant exhibits impaired growth in minimal medium under anaerobic conditions (Silva-Valenzuela et al., 2016), preventing the use of this strain to determine a role for ArcA on oxygen-modulated lipid A hydroxylation. Therefore, we decided to characterize the lipid A species profile when the gene dose of *arcA* is increased. To this end, we transformed the wild-type strain with a plasmid harboring *arcA* gene (parcA). Under aerobic conditions, ArcA overexpression led to an increase of non-hydroxylated lipid A species (*m/z* 1797.2 and 2035.8) together with a decrease of the hydroxylated counterparts (*m/z* 1812.9 and 2051.7; **Figure 1**, upper right panel). Of note, this distribution of lipid A species resembles that exhibited by the wild-type strain grown under anaerobic conditions (**Figure 1**, compare upper right and lower left panels). On the other hand, only non-hydroxylated lipid A species were detected when ArcA was overexpressed under anaerobic conditions (**Figure 1**, lower right panel). These results indicate that ArcA modulates lipid A hydroxylation in *S*. Enteritidis. Thus, the involvement of Fnr and ArcA transcription regulators in lipid A modification reveals a novel role for oxygen as a regulatory cue controlling this process in *S*. Enteritidis.

### *lpxO* Expression Is Regulated by Fnr and ArcA in Response to Oxygen Availability

**FIGURE 3:**
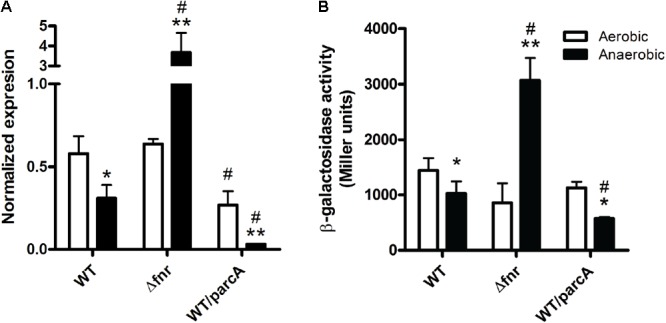
Expression of *lpxO* in *S*. Enteritidis strains. Expression levels were determined by qRT-PCR and normalized using *rpoD* as housekeeping gene **(A)** or by measuring β-galactosidase activity using strains carrying an *lpxO*–*lacZ* transcriptional fusion **(B)**. Bars represent mean values from three independent replicates. Error bars denote standard deviation. Statistical significance of observed differences was determined using a two-tailed Student’s *t*-test (^∗^*p* < 0.05 vs. the same strain grown aerobically; ^∗∗^*p* < 0.01 vs. the same strain grown aerobically; ^#^*p* < 0.05 vs. the wild-type strain grown under the same condition).

The above results led us to hypothesize that *lpxO* expression is regulated by oxygen availability through the activity of transcription regulators ArcA and/or Fnr. To address this notion, we measured the relative abundance of *lpxO* transcript by qRT-PCR in the wild-type strain, the Δ*fnr* mutant, and the wild-type strain overexpressing ArcA (wt/parcA) (**Figure 3A**). In addition, we analyzed the activity of the *lpxO* promoter using a chromosomal transcriptional fusion to *lacZ* in the same genetic backgrounds (**Figure 3B**). Both qRT-PCR and β-galactosidase assays revealed that *lpxO* expression in the wild-type strain is reduced under anaerobic conditions (**Figure 3**). This regulation could account for the reduction in lipid A hydroxylation levels observed in anaerobiosis (**Figure 1**, lower left panel). These results indicate that oxygen availability regulates *lpxO* expression.

On the contrary, *lpxO* expression in the Δ*fnr* mutant was increased under anaerobic conditions (**Figure 3**). Thus, the oxygen-dependent regulation of *lpxO* expression described for the wild-type strain is lost when *fnr* is deleted, indicating that Fnr is a negative regulator of *lpxO* expression under anaerobic conditions. This higher *lpxO* expression under anaerobiosis was not reflected on an increment in the relative abundance of lipid A hydroxylated species in this mutant grown under the same conditions (**Figure 1**, lower middle panel). This was expected considering that oxygen is a substrate of the LpxO-catalyzed reaction (Gibbons et al., 2000).

As in the case of the wild type, *lpxO* expression in the wt/parcA strain is strongly reduced in anaerobiosis (**Figure 3**), reflecting the absence of hydroxylated lipid A species detected by mass spectrometry analysis (**Figure 1**, lower right panel). Of note, *arcA* overexpression also accounts for lower *lpxO* expression under anaerobic conditions in the wt/parcA strain when compared to the wild-type strain (**Figure 3**). These observations suggest that ArcA is a negative regulator of *lpxO* expression under anaerobic conditions.

### Fnr and ArcA Bind to the *lpxO* Promoter Region

**FIGURE 4:**
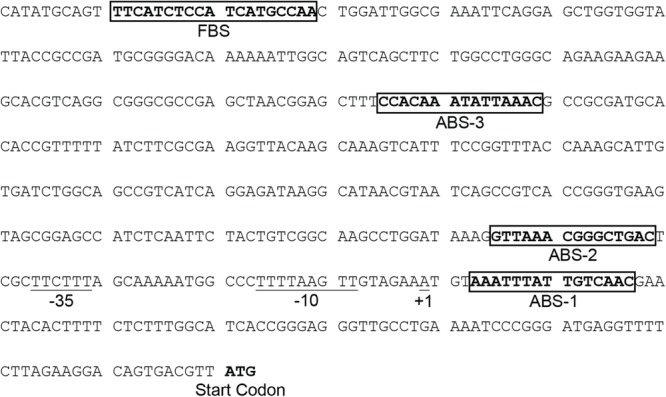
Nucleotide sequence of the *lpxO* promoter region in *S*. Enteritidis. The genome sequence of strain NCTC13349 was obtained from EMBL (accession number AM933172). Underlined sequences represent transcriptional start site (+1), –10, and –35 elements predicted by Softberry BPROM tool http://www.softberry.com/berry.phtml. The locations of FBS and ABS predicted by PRODORIC Virtual Footprint 3.0 server http://www.prodoric.de/vfp/ are shown in boxes.

**FIGURE 5:**
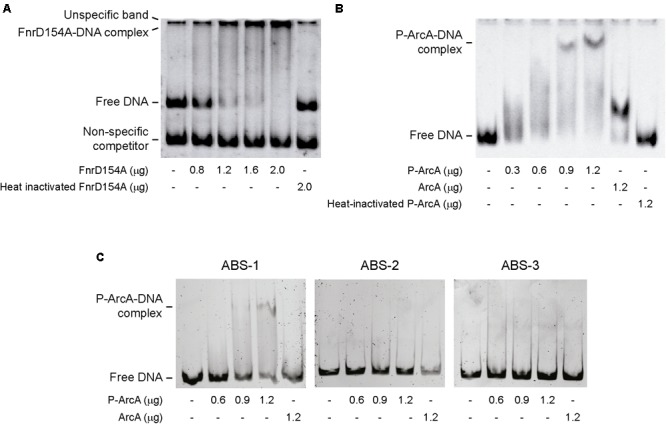
Binding of Fnr and ArcA to *S*. Enteritidis *lpxO* promoter region. EMSA were conducted using a 480 bp fragment carrying the complete *lpxO* promoter and purified FnrD154A **(A)** or P-ArcA **(B)** proteins. EMSA were also performed incubating purified P-ArcA with fragments of the *lpxO* promoter region containing ABS-1, ABS-2, or ABS-3 **(C)**. In all cases, EMSA included heat-inactivated proteins and/or non-phosphorylated ArcA as negative controls. When indicated, a 261 bp DNA fragment was used as non-specific competitor.

Our results indicate that Fnr and ArcA have a role controlling lipid A hydroxylation by modulating *lpxO* expression. However, it remains unknown whether the regulatory effect of these transcription factors is exerted directly or indirectly on the *lpxO* promoter. A bioinformatic analysis using the PRODORIC tool (Munch et al., 2005) allowed us to identify one putative Fnr binding site (FBS) and three putative ABS in the *lpxO* promoter region (**Figure 4**), suggesting a direct effect for both regulators. To confirm this notion, we performed electrophoretic mobility shift assays (EMSA) using a DNA fragment including the *lpxO* promoter and FnrD154A, a constitutively active variant of Fnr (Lazazzera et al., 1993), or P-ArcA. Our results showed that both FnrD154A and P-ArcA bound to the *lpxO* promoter in a dose-dependent fashion (**Figure 5**). When incubated in the presence of FnrD154A, the intensity of a highly retarded band on top of the gel increased considerably with increasing amounts of FnrD154 (**Figure 5A**). The mobility of this DNA–protein complex was similar to an unspecific band present in each lane. Concomitantly, we observed a pronounced decrease in the intensity of the band corresponding to free DNA probe. No binding of FnrD154A to a non-specific DNA competitor present in each reaction mix was detected. On the other hand, both P-ArcA and ArcA bound to the *lpxO* promoter, but generated different band-shift patterns (**Figure 5B**). The same behavior has been described for other ArcA-controlled promoters in *E. coli* (Lynch and Lin, 1996; Mika and Hengge, 2005). Of note, only in the presence of high amounts of P-ArcA (0.9 and 1.2 μg), the interaction with the *lpxO* promoter seems to be more stable and produced a complex with the slowest mobility (**Figure 5B**), which is consistent with P-ArcA multimerization (Jeon et al., 2001). As expected, no binding was observed to any DNA probe when either protein was heat-inactivated (**Figures 5A,B**). These results indicate that Fnr and ArcA regulate *lpxO* expression through direct binding to its promoter region.

Because the *lpxO* promoter only presents a unique FBS, this element should be responsible for Fnr binding to the promoter. To determine which putative ABS in the *lpxO* promoter is responsible for P-ArcA binding, the promoter region was dissected in three regions each one containing only one putative ABS. Our results showed that P-ArcA only bound to the *lpxO* promoter fragment containing ABS-1 (**Figure 5C**). In addition, no bound was detected when either fragment was incubated with non-phosphorylated ArcA. Noteworthy, ABS-1 is located downstream of the putative -10 element in a position that likely represses transcription.

## Discussion

In this work, we showed that oxygen is not only a substrate for LpxO, the enzyme that catalyzes lipid A hydroxylation, but also acts as an environmental cue that regulates this process in *S*. Enteritidis. In addition, we also demonstrate that global regulators Fnr and ArcA control the oxygen-dependent lipid A hydroxylation by directly regulating the expression of *lpxO*. To the best of our knowledge, this is the first time that a regulatory role for oxygen and Fnr and ArcA on lipid A covalent modifications has been described in *Salmonella*. Taking into consideration that oxygen is essential for lipid A hydroxylation, it makes perfect sense the oxygen-dependent regulation of *lpxO* expression in order to limit the production of LpxO during anaerobiosis, where one of its substrates is absent.

As described in *S*. Typhimurium (Gibbons et al., 2000), our data showed that lipid A hydroxylation is dependent on oxygen availability in *S*. Enteritidis (**Figure 1**). These observations are in line with the described role played by this molecule as LpxO substrate. Noteworthy, our results also revealed that *lpxO* expression is regulated by oxygen availability (**Figure 3**). Furthermore, this regulation correlates with the relative levels of hydroxylated and non-hydroxylated lipid A species produced by wild-type *S*. Enteritidis during aerobiosis and anaerobiosis (**Figure 1**). In contrast, oxygen-dependent regulation of lipid A hydroxylation was lost in the Δ*fnr* mutant and in the wt/parcA strain (**Figure 1**), which correlates with the altered expression of *lpxO* observed in these genetic backgrounds (**Figure 3**). Of note, the lipid A species distribution shown by strain wt/parcA grown aerobically resembles the one shown by the wild-type strain grown anaerobically (**Figure 1**). These observations further support the notion that oxygen is not only an LpxO substrate, but also constitutes a regulatory signal controlling lipid A hydroxylation.

Together, these findings suggested that the role played by Fnr and ArcA in lipid A hydroxylation most probably occurs by controlling *lpxO* expression. This idea was supported by our bioinformatics analyses that revealed the presence of a putative FBS centered at position -379, and three putative ABS centered at positions 12, -47, and -237 with respect to the predicted transcription initiation site of *lpxO* (**Figure 4**). Several genes regulated by oxygen availability in *E. coli*, such as *cydAB, cyoABCDE* (Salmon et al., 2005), *ydbN*, and *ydeJ* (Partridge et al., 2006), also possess similarly positioned ABS and/or FBS. Our results also showed that both Fnr and ArcA are able to bind *in vitro* to the promoter regions of *lpxO* (**Figure 5**), strongly suggesting a direct role for these regulators in the control of *lpxO* transcription. After assessing the functionality of each ABS in the *lpxO* promoter by EMSA, we observed that ArcA binds to ABS-1, but not to ABS-2 and ABS-3. Since each ABS was evaluated individually, we cannot rule out a cooperative interaction among these sites for ArcA binding to the *lpxO* promoter *in vivo*.

The regulation of *lpxO* expression has been scarcely studied. Just one work reported that a *S*. Typhimurium Δ*phoP* mutant presents wild-type levels of *lpxO* expression, indicating that this gene is not regulated by PhoP/PhoQ (Gibbons et al., 2005). In spite of this, the same study reported that *lpxO* expression was increased in a strain carrying the *pho-24* allele encoding a constitutively active version of PhoP. According to our bioinformatic analyses (data not shown), the *lpxO* promoter region does not contain putative PhoP binding sequences, suggesting that the observations made in the *pho-24* background are due to an indirect effect on *lpxO* expression.

Our data also indicate that Fnr not only controls lipid A hydroxylation, since several lipid A species not observed in the wild-type strain were detected in the Δ*fnr* mutant (**Figure 1**). Similarly, some of these species were also detected in a Δ*arcA* strain grown aerobically (Supplementary Figure S2). This indicates that Fnr and/or ArcA also control the expression of genes encoding other enzymes involved in lipid A covalent modifications. Supporting this idea, our unpublished bioinformatic analyses revealed putative binding sites for both regulators in the promoter regions of genes *eptA, lpxR*, and *pagP* (data not shown). Furthermore, we recently demonstrated an oxygen-dependent regulation of OAg chain-length distribution in *S*. Enteritidis (Silva-Valenzuela et al., 2016). This process was dependent on the regulation exerted by Fnr and ArcA on the expression of genes *wzz*_SE_ and *wzz*_fepE_, which encode the OAg chain length regulators. Thus, it is tempting to speculate that oxygen availability modulates LPS plasticity through these transcription regulators, contributing to the adaptation of *S*. Enteritidis to environmental challenges.

Although we can only speculate on the physiological role of the described oxygen-dependent control of lipid A hydroxylation, it has been established that most lipid A covalent modifications have an impact on the virulence of Gram-negative bacteria (reviewed in Needham and Trent, 2013). For instance, *K. pneumoniae* produces hydroxylated lipid A *in vivo* during lung infection in mice (Llobet et al., 2015). In addition, stimulation of alveolar and bone marrow-derived murine macrophages *in vitro* with hydroxylated lipid A from *K. pneumoniae* triggers a lower TLR-4-dependent inflammatory response than non-hydroxylated lipid A (Llobet et al., 2015; Mills et al., 2017). Furthermore, lipid A hydroxylation has been linked to *K. pneumoniae* resistance to CAMPs such as colistin and polymyxin B (Llobet et al., 2015; Mills et al., 2017). All these observations indicate that lipid A hydroxylation contributes to *K. pneumoniae* pathogenicity by facilitating evasion of the innate immune response.

Regarding *S*. Typhimurium, it has been reported that lipid A hydroxylation levels impact on epithelial cell invasion, intracellular survival in macrophages, and fitness during infection in mice (Moreira et al., 2013). Thus, these observations strongly suggest that appropriate levels of hydroxylated lipid A are essential for *Salmonella* virulence. However, it is important to mention that these observations were obtained from *in vitro* experiments and we still do not know whether *Salmonella* produces or not hydroxylated lipid A *in vivo*. In spite of this, it is known that non-typhoidal *Salmonella* serovars (including *S*. Enteritidis and *S*. Typhimurium, among others) trigger a strong inflammatory response in the gut during infection. This process generates a beneficial niche containing specific substrates that the pathogen can use in order to outgrowth the gut microbiota. Furthermore, the host inflammatory response enables the transmission of the pathogen by the fecal–oral route (revised in Rivera-Chávez and Bäumler, 2015). Taking into consideration that hydroxylation reduces the pro-inflammatory effects of lipid A in *K. pneumoniae*, we hypothesize that non-typhoidal *Salmonella* serovars mainly produce non-hydroxylated lipid A in response to anaerobic conditions in the gut, increasing the pro-inflammatory properties of the bacterial envelope in order to colonize the host efficiently. Further studies are required to confirm this hypothesis.

In addition to *Salmonella* and *Klebsiella*, many Gram-negative bacterial pathogens such as *Bordetella bronchiseptica, Pseudomonas aeruginosa, Legionella pneumophila, Acinetobacter baumannii*, and *Vibrio cholerae* also produce a lipid A modified with hydroxylated secondary acyl chains (Kulshin et al., 1991; Zähringer et al., 1995; Gibbons et al., 2000; Beceiro et al., 2011; Hankins et al., 2011; MacArthur et al., 2011; Llobet et al., 2015). In *V. cholera*, this modification has been associated with resistance to polymyxin B (Hankins et al., 2011). In the case of *P. aeruginosa*, it has been reported that modifications of lipid A structure, including hydroxylation, are required to alter the innate immune responses in order to achieve bacterial persistence and chronic infections, as in the case of cystic fibrosis patients (Moskowitz and Ernst, 2010). Thus, lipid A hydroxylation seems to be a conserved mechanism exploited by bacterial pathogens in order to modulate the innate immune response of the host during infection.

## Conclusion

Overall, our results support a model in which Fnr and ArcA control the expression of *lpxO* in *S*. Enteritidis to achieve a fine-tuning of lipid A hydroxylation levels. This regulatory mechanism could modulate the pro-inflammatory properties of the bacterial envelope in response to variations in oxygen levels during infection.

## Author Contributions

PF, FV, CS, and SÁ conceived and designed the experiments. PF, FV, HG-P, FA, JO, and SG performed the experiments. PF, FV, HG-P, FA, CS, and SÁ analyzed the data. PF, CS, and SÁ contributed with reagents, materials, and analysis tools, and wrote the paper. All authors read and approved the final manuscript.

## Conflict of Interest Statement

The authors declare that the research was conducted in the absence of any commercial or financial relationships that could be construed as a potential conflict of interest.

## Footnotes

1 www.mmass.org

## References

[B1] Altier C. (2005). Genetic and environmental control of *Salmonella* invasion. *J. Microbiol.* 43 85–92. 15765061

[B2] Beceiro A. Llobet E. Aranda J. Bengoechea J. A. Doumith M. Hornsey M. (2011). Phosphoethanolamine modification of lipid A in colistin-resistant variants of *Acinetobacter baumannii* mediated by the *pmrAB* two-component regulatory system. *Antimicrob. Agents Chemother.* 55 3370–3379. 10.1128/AAC.00079-11 21576434 PMC3122444

[B3] Bravo D. Silva C. Carter J. A. Hoare A. Álvarez S. A. Blondel C. J. (2008). Growth-phase regulation of lipopolysaccharide O-antigen chain length influences serum resistance in serovars of *Salmonella*. *J. Med. Microbiol.* 57(Pt 8) 938–946. 10.1099/jmm.0.47848-0 18628492

[B4] Caroff M. Novikov A. (2011). Micromethods for lipid A isolation and structural characterization. *Methods Mol. Biol.* 739 135–146. 10.1007/978-1-61779-102-4_12 21567324

[B5] Cherepanov P. P. Wackernagel W. (1995). Gene disruption in *Escherichia coli*: TcR and KmR cassettes with the option of Flp-catalyzed excision of the antibiotic-resistance determinant. *Gene* 158 9–14. 10.1016/0378-1119(95)00193-A 7789817

[B6] Compan I. Touati D. (1994). Anaerobic activation of *arcA* transcription in *Escherichia coli*: roles of Fnr and ArcA. *Mol. Microbiol.* 11 955–964. 10.1111/j.1365-2958.1994.tb00374.x 8022271

[B7] Coward C. Sait L. Cogan T. Humphrey T. J. Maskell D. J. (2013). O-antigen repeat number in *Salmonella enterica* serovar Enteritidis is important for egg contamination, colonisation of the chicken reproductive tract and survival in egg albumen. *FEMS Microbiol. Lett.* 343 169–176. 10.1111/1574-6968.12143 23551176

[B8] Dalebroux Z. D. Miller S. I. (2014). *Salmonella*e PhoPQ regulation of the outer membrane to resist innate immunity. *Curr. Opin. Microbiol.* 17 106–113. 10.1016/j.mib.2013.12.005 24531506 PMC4043142

[B9] Darveau R. P. Hancock R. E. (1983). Procedure for isolation of bacterial lipopolysaccharides from both smooth and rough *Pseudomonas aeruginosa* and *Salmonella typhimurium* strains. *J. Bacteriol.* 155 831–838. 6409884 10.1128/jb.155.2.831-838.1983 PMC217756

[B10] Datsenko K. A. Wanner B. L. (2000). One-step inactivation of chromosomal genes in *Escherichia coli* K-12 using PCR products. *Proc. Natl. Acad. Sci. U.S.A.* 97 6640–6645. 10.1073/pnas.120163297 10829079 PMC18686

[B11] Ellermeier C. D. Janakiraman A. Slauch J. M. (2002). Construction of targeted single copy *lac* fusions using lambda Red and FLP-mediated site-specific recombination in bacteria. *Gene* 290 153–161. 10.1016/S0378-1119(02)00551-6 12062810

[B12] Fink R. C. Evans M. R. Porwollik S. Vázquez-Torres A. Jones-Carson J. Troxell B. (2007). FNR is a global regulator of virulence and anaerobic metabolism in *Salmonella enterica* serovar Typhimurium (ATCC 14028s). *J. Bacteriol.* 189 2262–2273. 10.1128/JB.00726-06 17220229 PMC1899381

[B13] Folch J. Lees M. Sloane Stanley G. H. (1957). A simple method for the isolation and purification of total lipides from animal tissues. *J. Biol. Chem.* 226 497–509. 13428781

[B14] Gibbons H. S. Kalb S. R. Cotter R. J. Raetz C. R. (2005). Role of Mg^2+^ and pH in the modification of *Salmonella* lipid A after endocytosis by macrophage tumour cells. *Mol. Microbiol.* 55 425–440. 10.1111/j.1365-2958.2004.04409.x 15659161

[B15] Gibbons H. S. Lin S. Cotter R. J. Raetz C. R. (2000). Oxygen requirement for the biosynthesis of the S-2-hydroxymyristate moiety in *Salmonella typhimurium* lipid A. Function of LpxO, a new Fe^2+^/α-ketoglutarate-dependent dioxygenase homologue. *J. Biol. Chem.* 275 32940–32949. 10.1074/jbc.M005779200 10903325

[B16] Gibbons H. S. Reynolds C. M. Guan Z. Raetz C. R. (2008). An inner membrane dioxygenase that generates the 2-hydroxymyristate moiety of *Salmonella* lipid A. *Biochemistry* 47 2814–2825. 10.1021/bi702457c 18254598 PMC2709818

[B17] Guard-Petter J. (2001). The chicken, the egg and *Salmonella enteritidis*. *Environ. Microbiol.* 3 421–430. 10.1046/j.1462-2920.2001.00213.x 11553232

[B18] Gunn J. S. Ryan S. S. Van Velkinburgh J. C. Ernst R. K. Miller S. I. (2000). Genetic and functional analysis of a PmrA-PmrB-regulated locus necessary for lipopolysaccharide modification, antimicrobial peptide resistance, and oral virulence of *Salmonella enterica* serovar Typhimurium. *Infect. Immun.* 68 6139–6146. 10.1128/IAI.68.11.6139-6146.2000 11035717 PMC97691

[B19] Guo L. Lim K. B. Poduje C. M. Daniel M. Gunn J. S. Hackett M. (1998). Lipid A acylation and bacterial resistance against vertebrate antimicrobial peptides. *Cell* 95 189–198. 10.1016/S0092-8674(00)81750-X 9790526

[B20] Hankins J. V. Madsen J. A. Giles D. K. Childers B. M. Klose K. E. Brodbelt J. S. (2011). Elucidation of a novel *Vibrio cholerae* lipid A secondary hydroxy-acyltransferase and its role in innate immune recognition. *Mol. Microbiol.* 81 1313–1329. 10.1111/j.1365-2958.2011.07765.x 21752109 PMC3178793

[B21] Jeon Y. Lee Y. S. Han J. S. Kim J. B. Hwang D. S. (2001). Multimerization of phosphorylated and non-phosphorylated ArcA is necessary for the response regulator function of the Arc two-component signal transduction system. *J. Biol. Chem.* 276 40873–40879. 10.1074/jbc.M104855200 11527965

[B22] Kawasaki K. China K. Nishijima M. (2007). Release of the lipopolysaccharide deacylase PagL from latency compensates for lack of lipopolysaccharide aminoarabinose modification-dependent resistance to the antimicrobial peptide polymyxin B in *Salmonella enterica*. *J. Bacteriol.* 189 4911–4919. 10.1128/JB.00451-07 17483225 PMC1913436

[B23] Kiley P. J. Beinert H. (1998). Oxygen sensing by the global regulator, FNR: the role of the iron-sulfur cluster. *FEMS Microbiol. Rev.* 22 341–352. 10.1111/j.1574-6976.1998.tb00375.x 9990723

[B24] Kong Q. Six D. A. Liu Q. Gu L. Wang S. Alamuri P. (2012). Phosphate groups of lipid A are essential for *Salmonella enterica* serovar Typhimurium virulence and affect innate and adaptive immunity. *Infect. Immun.* 80 3215–3224. 10.1128/IAI.00123-12 22753374 PMC3418755

[B25] Kroger C. Dillon S. C. Cameron A. D. Papenfort K. Sivasankaran S. K. Hokamp K. (2012). The transcriptional landscape and small RNAs of *Salmonella enterica* serovar Typhimurium. *Proc. Natl. Acad. Sci. U.S.A.* 109 E1277–E1286. 10.1073/pnas.1201061109 22538806 PMC3356629

[B26] Kulshin V. A. Zähringer U. Lindner B. Jäger K. E. Dmitriev B. A. Rietschel E. T. (1991). Structural characterization of the lipid A component of *Pseudomonas aeruginosa* wild-type and rough mutant lipopolysaccharides. *Eur. J. Biochem.* 198 697–704. 10.1111/j.1432-1033.1991.tb16069.x 1904818

[B27] Lazazzera B. A. Bates D. M. Kiley P. J. (1993). The activity of the *Escherichia coli* transcription factor FNR is regulated by a change in oligomeric state. *Genes Dev.* 7 1993–2005. 10.1101/gad.7.10.1993 8406003

[B28] Llobet E. Martinez-Moliner V. Moranta D. Dahlstrom K. M. Regueiro V. Tomas A. (2015). Deciphering tissue-induced *Klebsiella pneumoniae* lipid A structure. *Proc. Natl. Acad. Sci. U.S.A.* 112 E6369–E6378. 10.1073/pnas.1508820112 26578797 PMC4655541

[B29] Lynch A. S. Lin E. C. (1996). Transcriptional control mediated by the ArcA two-component response regulator protein of *Escherichia coli*: characterization of DNA binding at target promoters. *J. Bacteriol.* 178 6238–6249. 10.1128/jb.178.21.6238-6249.1996 8892825 PMC178496

[B30] MacArthur I. Jones J. W. Goodlett D. R. Ernst R. K. Preston A. (2011). Role of *pagL* and *lpxO* in *Bordetella bronchiseptica* lipid A biosynthesis. *J. Bacteriol.* 193 4726–4735. 10.1128/JB.01502-10 21764941 PMC3165656

[B31] Maloy S. (1990). *Experimental Techniques in Bacterial Genetics.* Boston, MA: Jones & Bartlett.

[B32] Marolda C. L. Lahiry P. Vinés E. Saldías S. Valvano M. A. (2006). Micromethods for the characterization of lipid A-core and O-antigen lipopolysaccharide. *Methods Mol. Biol.* 347 237–252. 10.1385/1-59745-17072014

[B33] Mika F. Hengge R. (2005). A two-component phosphotransfer network involving ArcB, ArcA, and RssB coordinates synthesis and proteolysis of sigmaS (RpoS) in *E. coli*. *Genes Dev.* 19 2770–2781. 10.1101/gad.353705 16291649 PMC1283968

[B34] Miller J. (1972). *Experiments in Molecular Genetics.* Cold Spring Harbor, NY: Cold Spring Harbor Laboratory Press.

[B35] Mills G. Dumigan A. Kidd T. Hobley L. Bengoechea J. A. (2017). Identification and characterization of two *Klebsiella pneumoniae lpxL* lipid A late acyltransferases and their role in virulence. *Infect. Immun.* 85 e68–e17. 10.1128/IAI.00068-17 PMC5563558 28652313

[B36] Moreira C. G. Herrera C. M. Needham B. D. Parker C. T. Libby S. J. Fang F. C. (2013). Virulence and stress-related periplasmic protein (VisP) in bacterial/host associations. *Proc. Natl. Acad. Sci. U.S.A.* 110 1470–1475. 10.1073/pnas.1215416110 23302685 PMC3557018

[B37] Moskowitz S. M. Ernst R. K. (2010). The role of *Pseudomonas* lipopolysaccharide in cystic fibrosis airway infection. *Subcell. Biochem.* 53 241–253. 10.1007/978-90-481-9078-2_11 20593270 PMC2933746

[B38] Munch R. Hiller K. Grote A. Scheer M. Klein J. Schobert M. (2005). Virtual footprint and PRODORIC: an integrative framework for regulon prediction in prokaryotes. *Bioinformatics* 21 4187–4189. 10.1093/bioinformatics/bti635 16109747

[B39] Needham B. D. Trent M. S. (2013). Fortifying the barrier: the impact of lipid A remodelling on bacterial pathogenesis. *Nat. Rev. Microbiol.* 11 467–481. 10.1038/nrmicro3047 23748343 PMC6913092

[B40] Park B. S. Song D. H. Kim H. M. Choi B. S. Lee H. Lee J. O. (2009). The structural basis of lipopolysaccharide recognition by the TLR4-MD-2 complex. *Nature* 458 1191–1195. 10.1038/nature07830 19252480

[B41] Partridge J. D. Scott C. Tang Y. Poole R. K. Green J. (2006). *Escherichia coli* transcriptome dynamics during the transition from anaerobic to aerobic conditions. *J. Biol. Chem.* 281 27806–27815. 10.1074/jbc.M603450200 16857675

[B42] Raetz C. R. (2001). Regulated covalent modifications of lipid A. *J. Endotoxin. Res.* 7 73–78. 10.1177/09680519010070010201 11521087

[B43] Raetz C. R. Reynolds C. M. Trent M. S. Bishop R. E. (2007). Lipid A modification systems in Gram-negative bacteria. *Annu. Rev. Biochem.* 76 295–329. 10.1146/annurev.biochem.76.010307.145803 17362200 PMC2569861

[B44] Raetz C. R. Whitfield C. (2002). Lipopolysaccharide endotoxins. *Annu. Rev. Biochem.* 71 635–700. 10.1146/annurev.biochem.71.110601.135414 12045108 PMC2569852

[B45] Ravcheev D. A. Gerasimova A. V. Mironov A. A. Gelfand M. S. (2007). Comparative genomic analysis of regulation of anaerobic respiration in ten genomes from three families of gamma-proteobacteria (Enterobacteriaceae, Pasteurellaceae, Vibrionaceae). *BMC Genomics* 8:54 10.1186/1471-2164-8-54 PMC1805755 17313674

[B46] Reynolds C. M. Ribeiro A. A. McGrath S. C. Cotter R. J. Raetz C. R. Trent M. S. (2006). An outer membrane enzyme encoded by *Salmonella typhimurium lpxR* that removes the 3′-acyloxyacyl moiety of lipid A. *J. Biol. Chem.* 281 21974–21987. 10.1074/jbc.M603527200 16704973 PMC2702521

[B47] Rhen M. Dorman C. J. (2005). Hierarchical gene regulators adapt *Salmonella enterica* to its host milieus. *Int. J. Med. Microbiol.* 294 487–502. 10.1016/j.ijmm.2004.11.004 15790293

[B48] Rivera-Chávez F. Bäumler A. J. (2015). The pyromaniac inside you: *Salmonella* metabolism in the host gut. *Annu. Rev. Microbiol.* 69 31–48. 10.1146/annurev-micro-091014-104108 26002180

[B49] Roberts J. A. Sockett P. N. (1994). The socio-economic impact of human *Salmonella enteritidis* infection. *Int. J. Food Microbiol.* 21 117–129. 10.1016/0168-1605(94)90205-4 8155469

[B50] Salmon K. A. Hung S. P. Steffen N. R. Krupp R. Baldi P. Hatfield G. W. (2005). Global gene expression profiling in *Escherichia coli* K12: effects of oxygen availability and ArcA. *J. Biol. Chem.* 280 15084–15096. 10.1074/jbc.M414030200 15699038

[B51] Santiviago C. A. Reynolds M. M. Porwollik S. Choi S. H. Long F. Andrews-Polymenis H. L. (2009). Analysis of pools of targeted *Salmonella* deletion mutants identifies novel genes affecting fitness during competitive infection in mice. *PLoS Pathog.* 5:e1000477 10.1371/journal.ppat.1000477 PMC2698986 19578432

[B52] Sawers G. (1999). The aerobic/anaerobic interface. *Curr. Opin. Microbiol.* 2 181–187. 10.1016/S1369-5274(99)80032-0 10322162

[B53] Schmieger H. (1972). Phage P22-mutants with increased or decreased transduction abilities. *Mol. Gen. Genet.* 119 75–88. 10.1007/BF00270447 4564719

[B54] Shi Y. Latifi T. Cromie M. J. Groisman E. A. (2004). Transcriptional control of the antimicrobial peptide resistance *ugtL* gene by the *Salmonella* PhoP and SlyA regulatory proteins. *J. Biol. Chem.* 279 38618–38625. 10.1074/jbc.M406149200 15208313

[B55] Silva C. A. Blondel C. J. Quezada C. P. Porwollik S. Andrews-Polymenis H. L. Toro C. S. (2012). Infection of mice by *Salmonella enterica* serovar Enteritidis involves additional genes that are absent in the genome of serovar Typhimurium. *Infect. Immun.* 80 839–849. 10.1128/iai.05497-11 22083712 PMC3264302

[B56] Silva-Valenzuela C. A. Velásquez F. Peñailillo J. Garcias-Papayani H. Fernández P. Tobar P. (2016). O-antigen chain-length distribution in *Salmonella enterica* serovar Enteritidis is regulated by oxygen availability. *Biochem. Biophys. Res. Commun.* 477 563–567. 10.1016/j.bbrc.2016.06.074 27343553

[B57] Tsai C. M. Frasch C. E. (1982). A sensitive silver stain for detecting lipopolysaccharides in polyacrylamide gels. *Anal. Biochem.* 119 115–119. 10.1016/0003-2697(82)90673-X 6176137

[B58] Yi E. C. Hackett M. (2000). Rapid isolation method for lipopolysaccharide and lipid A from Gram-negative bacteria. *Analyst* 125 651–656. 10.1039/b000368i 10892021

[B59] Zähringer U. Knirel Y. A. Lindner B. Helbig J. H. Sonesson A. Marre R. (1995). The lipopolysaccharide of *Legionella pneumophila* serogroup 1 (strain Philadelphia 1): chemical structure and biological significance. *Prog. Clin. Biol. Res.* 392 113–139. 8524918

