# Supplementary material for: Fnr and ArcA Regulate Lipid A Hydroxylation in Salmonella Enteritidis by Controlling lpxO Expression in Response to Oxygen Availability
Source: Front Microbiol. 2018 Jun 8;9:1220. doi: 10.3389/fmicb.2018.01220 (PMC6002686; doi:10.3389/fmicb.2018.01220)
Supplement: Supplementary file 1 [file Table_1.PDF]

Table S1.- Primers used in this study.

**Primer name**	**Sequence**	**Description/Purpose**
WlpxO1	AAGGACAGTGACGTTATGTTCGCAGCAGTGCAGGCTGGAGCTGCTTC	Construction of Δ*lpxO* mutant
WlpxO2	TACCGCCGGCCGGCTTCAGAGGAGGCTCATATGAATATCCTCCTTAG	Construction of Δ*lpxO* mutant
lpxOF	TTTCATCTCCATCATGCCAA	Confirmation of Δ*lpxO* mutant
lpxOR	CGGATGGATAGACTCAAGGC	Confirmation of Δ*lpxO* mutant
Wfnr1	CAATTACGGCTTGAGCAGACCTATGATCCCGTGTAGGCTGGAGCTGCTTC	Construction of Δ*fnr* mutant
Wfnr2	GCTGTTTTCAATAGTAATATACTTACCTTTCATATGAATATCCTCCTTAG	Construction of Δ*fnr* mutant
fnrF	CGCCATGAAGGCTATCTTTTATTAT	Confirmation of Δ*fnr* mutant
fnrR	CAGAAGATAACATCAATGGTTTAGC	Confirmation of Δ*fnr* mutant
lac	GACCATTTTCAATCCGCA	Confirmation of pKG136 single-copy integration
kan	TTTCTAGAGCTGTTAAAAGGACA	Confirmation of pKG136 single-copy integration
qlpxOF	ACGGCATTTCCAGAGTCGCA	Quantification of *lpxO* transcript levels using qRT-PCR
qlpxOR	GGCATAGGGGTCGCGATGTT	Quantification of *lpxO* transcript levels using qRT-PCR
qrpoDF	TGAAGTGTTCAAACAGTTCCGTCT	Quantification of *rpoD* transcript levels using qRT-PCR
qrpoDR	AGACCGGTCTCTTCTTCAATCTGC	Quantification of *rpoD* transcript levels using qRT-PCR
arcAF	TGCAAATTTGTGATGAAAGC	Cloning of *S*. Enteritidis *arcA* (promoter included) in pBAD-TOPO
arcAR	AAAGAGTACGTCATAACGGC	Cloning of *S*. Enteritidis *arcA* (promoter included) in pBAD-TOPO

arcA_PF	TAAGAGGAATAATAAATGCAGACCCCGC	Cloning of *S*. Enteritidis *arcA* ORF in pBAD-TOPO
arcA_PR	ATCCTGCAGGTCGCCGCAGA	Cloning of *S*. Enteritidis *arcA* ORF in pBAD-TOPO
fnr_PF	TAAGAGGAATAATAAATGATCCCGGAAA	Cloning of *E. coli* K12 *fnrD154A* ORF in pBAD-TOPO
fnr_PR	AGCGACGTTGCGGGTATGAC	Cloning of *E. coli* K12 *fnrD154A* ORF in pBAD-TOPO
pBADF	ATGCCATAGCATTTTTATCC	Confirmation of PCR product cloned in pBAD-TOPO
pBADR	GATTTAATCTGTATCAGG	Confirmation of PCR product cloned in pBAD-TOPO
lpxO_up1F*	AGTTTCATCTCCATCATGCC	Amplification of the *lpxO* promoter region including ABS-1
lpxO_up1R	TGGCCCTTTTAAGTTGTAGA	Amplification of the *lpxO* promoter region including ABS-1
lpxO_up2F	AAAGCATTGTGATCTGGCAG	Amplification of the *lpxO* promoter region including ABS-2
lpxO_up2R	CAACTTAAAAGGGCCATTTT	Amplification of the *lpxO* promoter region including ABS-2
lpxO_up3F	GTAACCTTCGCGAAGATAAA	Amplification of the *lpxO* promoter region including ABS-3
lpxO_up3R*	TCTAAGAAAACCTCATCCCG	Amplification of the *lpxO* promoter region including ABS-3
SP6	TACGATTTAGGTGACACTATAG	Amplification of pGEM-T Easy polylinker region
T7	TAATACGACTCACTATAGGG	Amplification of pGEM-T Easy polylinker region

* Primers lpxO_up1F and lpxO_up3R were also used to amplify the complete promoter region of *lpxO*, which includes ABS-1, ABS-2 and ABS-3.

Underlined sequences correspond to the region that anneals to the 5’ or 3’ end of the antibiotic-resistance cassette in template vectors pCLF3 and pCLF4.
