# Supplementary material for: Fnr and ArcA Regulate Lipid A Hydroxylation in Salmonella Enteritidis by Controlling lpxO Expression in Response to Oxygen Availability
Source: Front Microbiol. 2018 Jun 8;9:1220. doi: 10.3389/fmicb.2018.01220 (PMC6002686; doi:10.3389/fmicb.2018.01220)
Supplement: Supplementary file 2 [file Image_1.PDF]

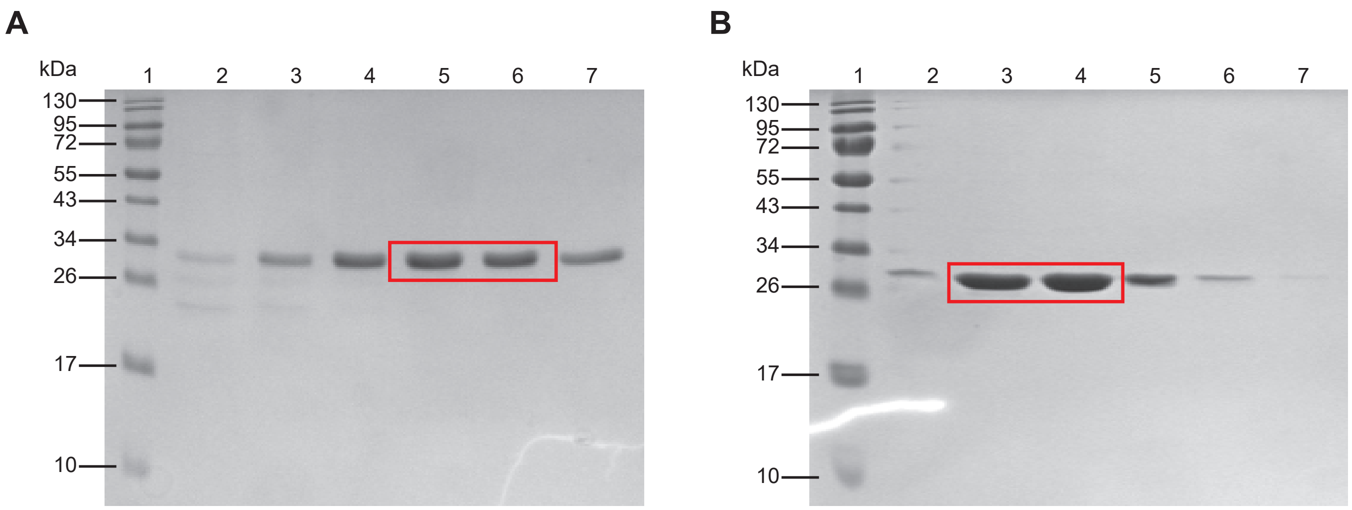

Figure S1.- SDS-PAGE analysis of recombinant FnrD154A and ArcA purified by nickel-affinity chromatography. Eluted fractions obtained from FnrD154A (A) or ArcA (B) purifications were resolved in 12% polyacrylamide gels and stained with Coomassie Brilliant Blue G-250 (lanes 2-7). In each case, a pool of the eluted fractions highlighted in boxes were used for EMSA. PageRuler Prestained Protein Ladder (Thermo Scientific) was used as molecular weight standard (lane 1).
