# Supplementary material for: Fnr and ArcA Regulate Lipid A Hydroxylation in Salmonella Enteritidis by Controlling lpxO Expression in Response to Oxygen Availability
Source: Front Microbiol. 2018 Jun 8;9:1220. doi: 10.3389/fmicb.2018.01220 (PMC6002686; doi:10.3389/fmicb.2018.01220)
Supplement: Supplementary file 3 [file Image_2.pdf]

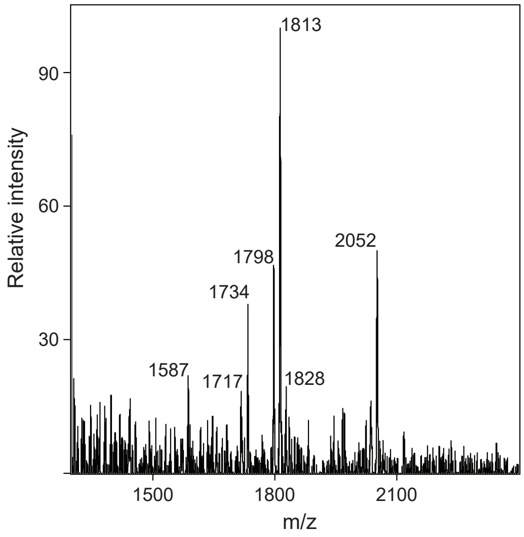

Figure S2.- Mass spectrometry analysis of lipid A species produced by *S*. Enteritidis ∆*arcA* mutant. Lipid A samples were obtained from cultures of a ∆*arcA* mutant (Silva-Valenzuela et al., 2016. doi: 10.1016/j.bbrc.2016.06.074) grown under aerobic conditions. MALDI-TOF mass spectrometry analysis of lipid A preparations was performed and experimental m/z values are indicated.
